# 2D- and 3D-cultures of human trabecular meshwork cells: A preliminary assessment of an in vitro model for glaucoma study

**DOI:** 10.1371/journal.pone.0221942

**Published:** 2019-09-06

**Authors:** Stefania Vernazza, Sara Tirendi, Sonia Scarfì, Mario Passalacqua, Francesco Oddone, Carlo Enrico Traverso, Ilaria Rizzato, Anna Maria Bassi, Sergio Claudio Saccà

**Affiliations:** 1 IRCCS, Fondazione G.B. Bietti, Rome, Italy; 2 Department of Experimental Medicine (DIMES), University of Genoa, Genoa, Italy; 3 Inter-University Center for the Promotion of the 3Rs Principles in Teaching & Research (Centro 3R), Italy; 4 Department of Earth, Environment and Life Sciences (DISTAV), University of Genoa, Genoa, Italy; 5 Department of Neuroscience, Rehabilitation, Ophthalmology, Genetics, Maternal and Child Health (DINOGMI), University of Genoa, Genoa, Italy; 6 Department of Modern Languages and Cultures (LCM), University of Genoa, Genoa, Italy; 7 IRCCS, San Martino General Hospital, Ophthalmology Unit, Genoa, Italy; Oregon Health and Science University, UNITED STATES

## Abstract

A physiologically relevant *in vitro* human-based model could be the ‘gold standard’ to clarify the pathological steps involved in glaucoma onset. In this regard, human 3D cultures may represent an excellent starting point to achieve this goal. Indeed, the 3D matrix allows to re-create the in vivo-like tissue architecture, maintaining its functionality and cellular behaviour, compared to the 2D model. Thus, we propose a comparison between the 2D and 3D in vitro models of human trabecular meshwork cells in terms of cellular responses after chronic stress exposure. Our results showed that 3D-cells are more sensitive to intracellular reactive oxidative specie production induced by hydrogen peroxide treatment, compared to 2D cultures. Additionally, in 3D cultures a more accurate regulation of the apoptosis trigger and cell adaptation mechanisms was detected than in 2D models. In line with these findings, the 3D-HTMC model shows the ability to better mimic the in vivo cell behaviour in adaptive responses to chronic oxidative stress than 2D.

## Introduction

Glaucoma is a progressive optic neuropathy that leads to irreversible blindness. From a molecular point of view it is possible to define glaucoma as a disease able to develop the pro-apoptotic signals heading for the optic nerve head. In its pathogenesis a central role is played by the trabecular meshwork (TM) that together with the inner wall of the Schlemm’s canal forms the conventional outflow path of the aqueous humor [1]. Its progressive functional and morphological impairment leads to the increase of intraocular pressure (IOP) [2]. The *primum movens* is probably due to the oxidative damage that triggers a significant cascade of events leading to different death pathways, such as apoptosis and/or autophagy of the retinal ganglion cells (RGCs) and to their functional deterioration [3–5]. However, there are many other aspects related to oxidative stress in glaucoma such as cellularity or the appearance of senescence or autophagy, all contributing to the development of glaucomatous disease. Moreover This explains why, although the IOP is the only risk factor that can be treated, many other elements must be understood in order to counter this multifactorial disease. Indeed, a wide variety of animal models such as naturally-occurring and induced models have been developed in order to understand the effect of elevated IOP on animal RGCs loss (i.e. Laser Photocoagulation of the Perilimbal Region, autologous fixed red blood cell injection or microbead injection into the anterior chamber, hypertonic saline injection into the episcleral veins, and so on). However, to reach the gold standard and to clarify the onset and the pathological course of the disease, it is necessary to improve in vitro human-based models to provide an accurate analysis of cell behaviour and of the molecular mechanisms which are involved in glaucoma [6–8]. As is well-known, 3D cultures represent the starting point for reliable *in vitro models* because, unlike 2D cultures, they preserve the physiological relevance of in vivo conditions [9]. Indeed, cells grown in 2D on plastic surfaces are unable to simulate real in vivo conditions because of the lack of cell shape and geometry [10]. However, the static culture conditions in both models represents a limiting factor for transporting the necessary oxygen and nutrients to maintain cell viability and function [11]. In our opinion, ex vivo − i.e., human cell-based − studies can be more effective in understanding the molecular mechanisms that underlie glaucoma disease, compared to animal-based glaucoma models. Nowadays, 3D technology is constantly evolving as it is widely used in several fields such as oncology, toxicology and drugs screening or to mimic in vivo tissue by tissue engineering [9, 12–16]. The aim of the present study is to assess 2D and 3D *in vitro* models of human trabecular meshwork cells (HTMC), in order to verify which can better mimic the TM impairment observed in glaucoma patients, and therefore be considered as a reliable starting point for glaucoma research. The TM is comparable to a real organ rather than a tissue, whose functionality can be influenced by several stimuli. In particular, oxidative stress seems likely to trigger the impairment of TM [2].

Taking into account that an oxidative stress condition can arise slowly, and extend over time, in our study 2D and 3D-HTMC cultures were exposed up to 72 hours to a sub-toxic dose of the oxidative stressor hydrogen peroxide, H_2_O_2_ to verify if different culture growth conditions can influence the cellular response to such stimulus. The effect of H_2_O_2_ exposure in HTMC was verified in terms of reactive oxygen species (ROS) production, changes of cell morphology, influence on healthy/metabolic state, as well as triggering of inflammation and apoptosis.

## Materials and methods

### Cell cultures

Human trabecular meshwork cells (HTMC) and Trabecular Meshwork Growth Medium (TMGM) came from Cell APPLICATION INC. (San Diego, CA, USA). According to consensus recommendations reported by Keller et al. [17], Cell APPLICATION laboratory gave the official report on the evidence that HTMC cells, made available by their Company, express several markers related to a trabecular phenotype, since the HTMC resulted responsive to Dexamethasone treatment by increased protein level expression of fibronectin, α-smooth muscle actin, myocilin and the cross-linked actin networks (CLANs) (see: https://www.cellapplications.com/human-trabecular-meshwork-cells-htmc and S1 File Cell Application Inc. further information).

HTMCs were grown routinely as monolayers (2D) in standard TMGM.

HTMC were maintained at 37°C in a humidified atmosphere containing 5% CO_2_, according to the manufacturer’s indications. Cells were sub-cultured by TripLE^™^ Express (Invitrogen Life Technologies) treatment when the original flask was approximately 75% confluent. All cell cultures were found to be mycoplasma-free during regular checks with the Reagent Set Mycoplasma Euroclone (Euroclone^®^ Milan, Italy).

2D and 3D-HTMC were seeded at 4 x 10 ^4^/cm^2^ and 2.5 x 10 ^5^/cm^3^ cells, respectively, in Primo^®^ TC Flask 25 cm^2^, and 96-wells plate (Euroclone^®^, Milano Italy), in BD-Falcon^TM^ cultures Slides (BD Biosciences Europe, Erembodegem, Belgium) and in Millipore TC-Plate 24-wells (Merck S.p.a., Merck KGaA, Darmstadt, Germany).

3D cultures were performed by embedding HTMC into Corning^®^ Matrigel^®^ Matrix (Corning Life Sciences, Tewksbury, MA USA)

Briefly, a suspension of 500,000 HTMCs was slightly centrifuged for 5 min at 90 rcf, and after removing the supernatant, the cell pellet was gently resuspended in 200 μl of undiluted Matrigel^™^ at 4°C. The embedded HTMCs were then gently transferred by pipette into culture chambers (1.9 cm^2^ growth area/dish) and the culture medium was added (1ml/dish) after 15 min, which is the necessary time for the Matrigel to reach its gelling state and its thickness resulted to be 1.13 mm, by applying the following formula: *Matrigel volume (0*.*2 ml) /growth area*.

(dx.doi.org/10.17504/protocols.io.574g9qw)

### Experimental conditions

Before performing experimental treatments, in order to reduce any Fetal Bovine Serum (FBS) interference on cellular proliferation, 2D and 3D HTMC cultures were maintained for at least 24 hrs in low and high glucose DMEM (1:1 mix), 2mM L-glutamine, 0.5% gentamicin and 100μg/ml streptomycin, w/o (FBS) [17]. Chronic stress exposure was performed in 2D- and 3D-HTMC up to 72 hrs, by daily exposure for 2 hrs to 500 μM H_2_O_2_, with 22-hour recovery phases in between, according to Poehlmann et al., 2013 and Kaczara et al., 2010 [18,19]. The H_2_O_2_ concentration was selected on the basis of previous experiments to identify a dose that resulted subtoxic, with a MTT viability index > 75% versus untreated cultures.

At the end of the experimental time 2D and 3D HTMCs were harvested by TripLE^™^ Express and from 3D Corning Cell Recovery (Corning Life Sciences B.V., Amsterdam, NL), respectively.

### Confocal analysis

At each selected check point time untreated and treated 2D- and 3D-HTMC, were set in 4% paraformaldehyde and permeabilized with 0.3% Triton X-100 (Sigma Aldrich®, Milan, Italy). Nuclei were stained with To-Pro^TM^ -3 Iodide 642/641(Thermo Fisher Scientific Inc., Monza, Italy), actin cytoskeleton was visualized using Phalloidin Alexa Fluor 488 (Cell Signaling Technology, Danvers, MA, USA). Fluorescence signals were captured at 60X magnification, by Leica TSC SP microscope (Leica Microsystems, Wetzlar, Germany) and elaborated by Fiji-ImageJ software, an open-source platform for biological-image analysis. Signals from different fluorescent probes were taken in sequential scan settings (3D reconstruction images).

### DCF assay

The monitoring of ROS production was performed by using the dichlorofluorescein (DCF) assay. HTMC 2D and 3D cultures were exposed to non-fluorescent 2',7'-dichlorodihydrofluorescein diacetate (H2DCFDA, Thermo Fisher Scientific Inc.), that freely permeates the plasma membrane and is reduced to the highly fluorescent 2',7'-dichlorofluorescein [20]. Experiments were performed in 96-well plates, and each condition analysed 6 times. Cells were seeded in 2D culture medium or in 3D Matrigel as already described at 10,000 cells/well the day before. After removing the culture medium, HTMCs were briefly rinsed with Hank’s balanced salt solution (HBSS) with calcium and magnesium and incubated with 10 μM H2DCFDA in HBSS at 37°C in 5% CO_2_ for 45 min. Then the H2DCFDA solution was removed and cultures were washed with HBSS and exposed to 500μM H_2_O_2_ in order to evaluate the ROS production over time in both culture models. DCF emission was determined at 1hr, 2hr and 4hr, on a fluorescent plate reader, at excitation and emission wavelengths of 485 and 520 nm, respectively. The fluorescence intensity was extrapolated after subtracting the blank (medium plus DFC for 2Ds and Matrigel plus medium plus DCF for 3Ds) and was expressed as percentage of relative fluorescence unit of treated vs untreated HTMC cultures.

### MTT assay

At the end of each experimental treatment, the cell viability in terms of mitochondria functionality was assessed in 2D and 3D models by the MTT assay [21]. The optical densities (OD) of the dissolved formazan crystals was determined spectrophotometrically at 570 nm. The quantification of cell viability was obtained by comparing the optical density of the extracts, and relative cell viability was calculated for each tissue as Arbitral Unit (AU), extrapolated by Optical Density (OD) of the samples.

### Alamar blue assay

The 2D- and 3D-HTMC healthy state, in terms of their metabolic activity, was measured by the Alamar Blue^TM^ (Invitrogen^TM^, Thermo Fisher Scientific Inc.) assay daily, during the last 4 hrs before the end of the 22 hrs of recovery time, by adding 10% Alamar Blue^TM^ directly in culture medium. The assay was carried out according to the manufacturer’s instructions. At each check point time, the resazurin reduction was extrapolated spectrophotometrically by monitoring absorbance at 570 and 630 nm wavelength. The results were expressed as AU extrapolated by OD of each sample.

### Human apoptosis array C1

Apoptosis was investigated by the semi-quantitative detection of 43 human apoptotic proteins, on customized Human Apoptosis Array C1 chip (RayBio^®^; Norcross, GA, USA) (**Table 1**). Briefly, after cell lysis each sample was incubated with antibody array membrane ON at 4°C. The day after, each membrane, after repeated washings with the Wash Buffer I and II (provided with the kit), was incubated with biotinylated antibody cocktail for 2 hrs at RT. Then, after washings with the abovementioned Wash Buffers, membranes were incubated with HRP-Streptavidin for 2 hrs at RT. After further washings, each membrane was incubated with detection buffer and the chemioluminescence was detected on a radiographic plate. The intensity of protein array signals was analysed using a BIORAD Geldoc 2000 and each protein spot was normalized against Positive Control Spots printed on each membrane.

**Table 1:** The mini map of Human Apoptosis Array C1 (according to RayBio^®^ manufacturer manual).

	A	B	C	D	E	F	G	H	I	J	K	L	M	N
**1**	POS	POS	NEG	NEG	Blank	Blank	bad	bax	Bcl2	Bcl2-w	BID	BIM	Caspase3	Caspase8
**2**														
**3**	CD40	CD40L	cIAP2	CytoC	DR6	Fas	FasL	Blank	Hsp27	Hsp60	Hsp70	HTRA2	IGF1	IGF2
**4**														
**5**	IGFBP1	IGFBP2	IGFBP3	IGFBP4	IGFBP5	IGFBP6	IGF-1R	Livin	P21	P27	P53	SMAC	Survivn	TNF RI
**6**														
**7**	TNF RII	TNFα	TNFβ	TRAIL R1	TRAIL R2	TRAIL R3	TRAIL R4	XIAP	Blank	Blank	NEG	NEG	POS	POS
**8**														

The data analysis was conducted following the Protocol directions of Human Apoptosis Array C1. The raw numerical densitometry data were subtracted from background (Negative Control signals) and normalized to the Positive Control signals. To determine the relative protein expression on different arrays, relating to untreated and treated samples, the algorithm according to Human Apoptosis Array C1 protocol was used.

### RNA extraction, cDNA synthesis and qPCR analyses

After experimental treatments, HTMC were subjected to gene expression profile by qPCR analysis and compared to control cells. Total RNA was extracted using the RNeasy Micro Kit (Qiagen S.r.l., Milan, Italy) according to the manufacturer's instructions. Quality and quantity of RNA was analysed using a NanoDrop spectrophotometer (Nanodrop Technologies, Wilmington, DE, USA). The cDNA (150 ng per sample) was synthesized by using SuperScript^TM^ III First Strand Synthesis System (Thermo Fisher Scientific). Each PCR reaction was performed in 10 μl containing: 5× HOT FIREPol^®^ EvaGreen^®^ qPCR Mix Plus (Solis BioDyne, Tartu, Estonia), 0.2 μM of each primers and 1 ng of synthesized cDNA. All samples were analysed in triplicate. The following thermal conditions were used: initial denaturation/hot start for 15 min, followed by 45 cycles with denaturation at 95° C for 15 s, annealing and elongation at 60° C for 60 s. The fluorescence was measured at the end of each elongation step. The next step was a slow heating (1° C/ s) of the amplified product from 55° C to 92° C in order to generate a melting temperature curve. Values were normalized to ubiquitin (reference gene) mRNA expression. All primers (**Table 2**), were designed using the Beacon Designer 7.0 software (Premier Biosoft International, Palo Alto CA, USA) and obtained from TIB MOLBIOL (Genoa, Italy). Data analyses were obtained using the DNA Engine Opticon^®^ 3 Real-Time Detection System Software program (3.03 version) and, in order to calculate the relative gene expression compared to an untreated (control) calibrator sample, the comparative threshold Ct method [22] was used within the Gene Expression Analysis for iCycler iQ Real Time Detection System software (Bio-Rad) [23]. Data are means ± S.D. of at least three independent experiments performed in triplicate.

**Table 2:** Primer sequences used for real time quantitative polymerase chain reaction analysis.

GENE	GeneBank	Forward	Reverse
IL-1α	NM_000575.4	CAATCTgTgTCTCTgAgTATC	TCAACCgTCTCTTCTTCA
IL1β	NM_000576.2	TgATggCTTATTACAgTggCAATg	gTAgTggTggTCggAgATTCg
IL6	NM_001318095.1	CAgATTTgAgAgTAgTgAggAAC	CgCAgAATgAgATgAgTTgTC
Ubiquitin C	NM_021009.7	ATTgggTCgCAgTTCTTg	TgCCTgACATTCTCgATggT

### Western blotting

Cell lysates were collected using RIPA buffer (Sigma Aldrich S.r.l., Milan, Italy) plus protease inhibitor cocktail (Complete Tablets, Roche Diagnostic GmbH, Mannheim Germany) and sonicated until solubilized. 25 μg of proteins were resolved in Ani kD^TM^ mini precast gel (Bio-Rad Laboratories, Inc., Hercules, CA, USA) in SDS-PAGE Running Buffer and transferred onto PVDF membrane (Thermo Scientific, Rockford, USA) and probed with primary antibodies against rabbit phospho-NF-kB p65, Ser 536 (Cell Signaling Technology) and mouse GAPDH (Santa Cruz Biotechnology, Santa Cruz, CA, USA) followed by incubation with HRP-conjugated secondary antibodies (NA9340V and NA931V, against rabbit and mouse primary antibodies, respectively, Amersham Life Science, Milan, Italy). The proteins were detected by Western Bright^TM^ ECL (Advansta, CA, USA), exposed to film and analysed using a BIORAD Geldoc 2000. The data presented were calculated after normalization with GAPDH. Densitometrical data obtained from Quantity One software (Bio-Rad) were applied for statistical analysis and normalized against the housekeeping GAPDH. The results were expressed as fold vs untreated cultures, respectively.

### Statistical analysis

Reported data are means ± standard deviation of the mean of three independent experiments performed in duplicate/triplicate. Significance was assessed by two-way ANOVA followed by Bonferroni’s test for DCF assay, MTT test, Alamar Blue assay, qPCR and NF-kB protein levels, and by one-way ANOVA followed by Bonferroni’s test for Apoptosis array using GraphPad Prism for Windows- version 5.03 and GraphPad Software, Inc., La Jolla, CA, USA). Statistically significant differences were set at p<0.05; p<0.01; p<0.001.

## Results

### Confocal analysis

**Fig 1:**
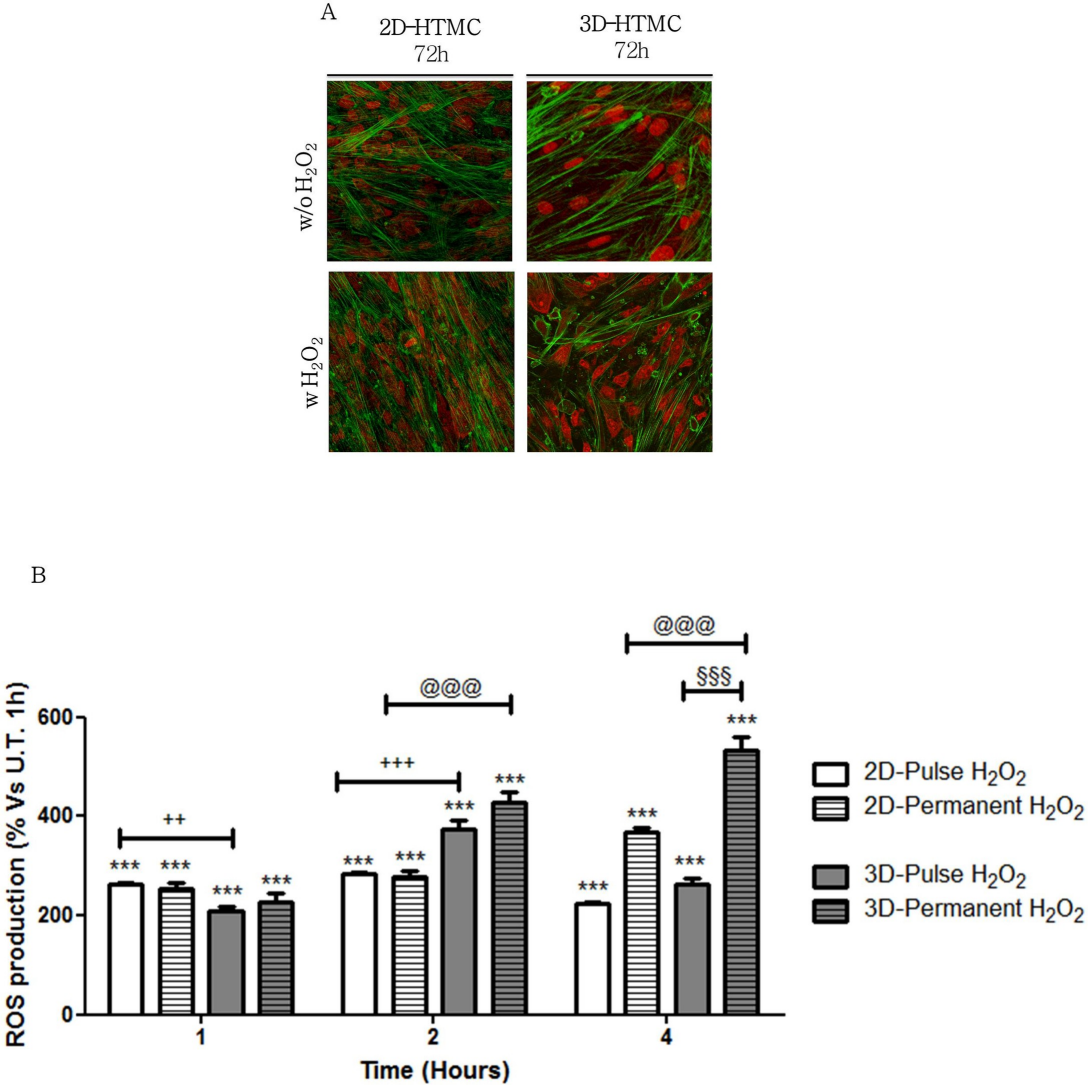
2D- and 3D-HTMC oxidative stress sensitivity. Panel A- Confocal microscopy analyses of nucleus and cytoskeletal markers were performed on untreated and H_2_O_2_-treated 2D- and 3D HTMC cells after 72 hrs of experimental procedures. Representative images are related to immunoreactivity for To-Pro^TM^ and Phalloidin, as nuclear and cytoskeleton markers, respectively. Merged images showed cytoskeleton plus Nucleus. Fluorescence signals were captured at 60x magnification. Panel B- DCF assay. DCF assay was performed on untreated and H_2_O_2_ (U.T.)-treated 2D- and 3D HTMC cells and the fluorescence was recorded at 1, 2 and 4 hrs of experimental procedures. Data are expressed as % of ROS production in untreated HTMCs after 1hr and represent the mean ± SD of 3 independent experiments, each performed six times. White and grey bars = 2D and 3D cultures, respectively ***p <0.001 vs respective untreated cultures (1h); §§§p<0.001 3D-permanent H_2_O_2_ vs 3D-pulse H_2_O_2_; @@@p<0.001 3D permanent H_2_O_2_ vs 2D permanent H_2_O_2_; ++p<0.01, +++p<0.001 3D pulse H_2_O_2_ vs 2D pulse H_2_O_2_ (Two-way ANOVA followed by Bonferroni posttest).

Spatial organization of 2D- and 3D-HTMC cultures was checked by confocal microscopy at the end of the experimental conditions. The merged images, obtained by untreated and H_2_O_2_-treated HTMC cultures, did not show any visible changes either in F-actin or in the nuclei structures of the two models. However, from a qualitative point of view, 2D-HTMCs showed a reduction of cell-to-cell interaction compared to the 3D model, in which cells showed an oblong morphology and better-ordered distribution in the matrix with a close resemblance to the in vivo physiological cell shape and an abundance of natural fibrous tissues (Fig 1 panel A).

### DCF assay for oxidative stress evaluation

The fluorometric DCF assay was performed in order to evaluate the ability of hydrogen peroxide to induce ROS production in the 2D- and 3D-HTMC cultures. The effect of hydrogen peroxide was investigated, either keeping it or not keeping it in HTMC culture medium, beyond the two hours. In both HTMC models the ROS production was therefore quantified at 1, 2 and 4 hrs (Fig 1 panel B). A different ROS production between 2D- and 3D- HTMCs over time was already observed after 1h of H_2_O_2_ exposure, in fact, the HTMCs cultured in 2D increased their intracellular ROS production by about 258%, while those cultured in 3D showed an increase by 210%, compared to their respective U.T. HTMC cultures. However, in the following time intervals (2 and 4 hrs), 3D-HTMCs showed a ROS production greater than 142%, after 2hrs, and 145%, after 4hrs, compared to the 2D model. Moreover, even after removal of the pro oxidant stimulus at 2h, the ROS production recorded at 4h, in 3D-HTMCs, was still higher than 2D- by about 116%.

### Mitochondrial respiratory functionality

**Fig 2:**
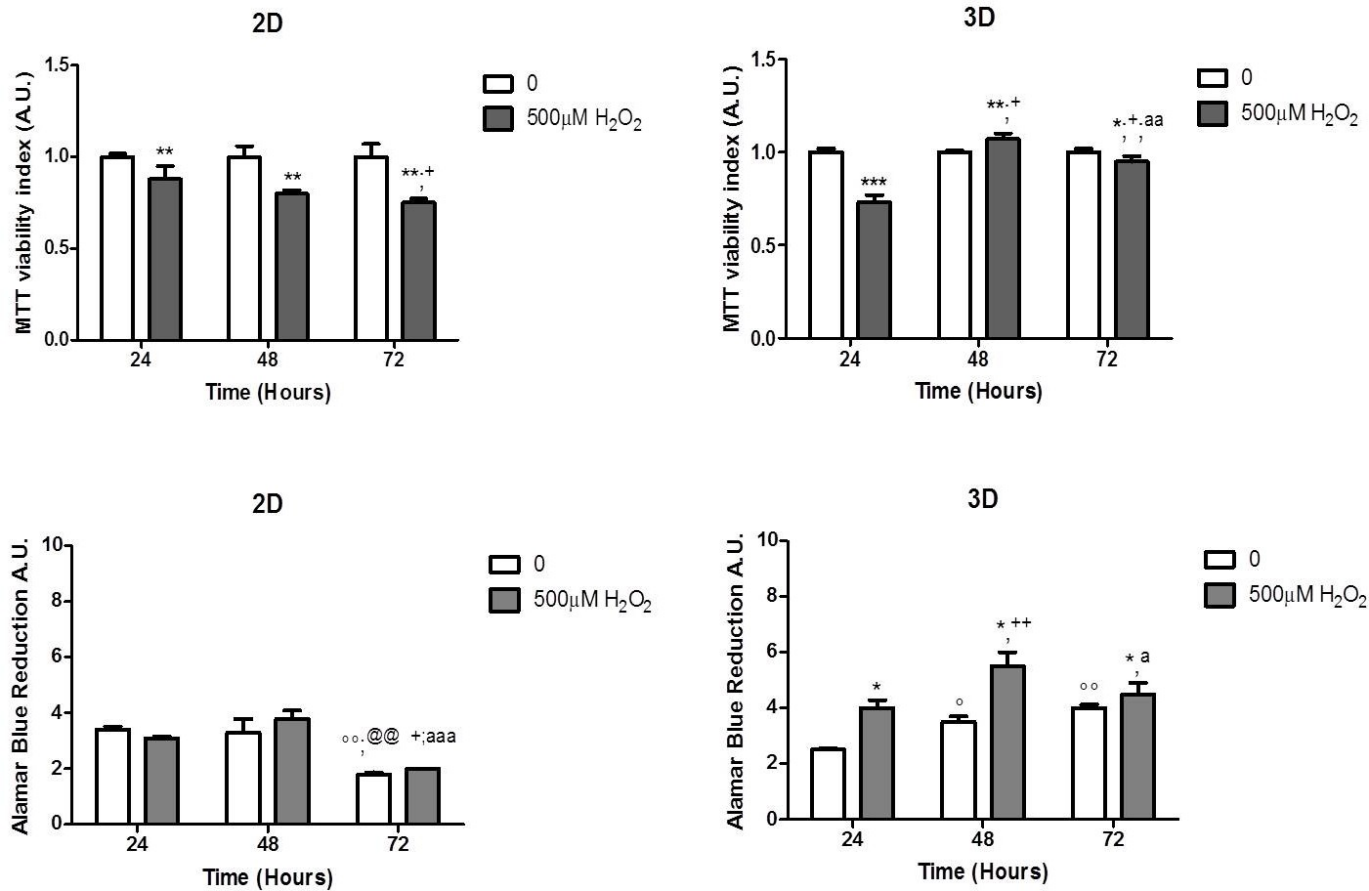
Effects of chronic H_2_O_2_ treatment on HTMC metabolic activity. Panel A- Mitochondrial respiratory functionality. MTT assay was performed in 2D- and 3D-HTMC exposed to H_2_O_2_ (500μM) for 24, 48 and 72 hrs. Panel B- Metabolic state of untreated and H_2_O_2_ treated 2D and 3D HTMC cultures, during experimental treatments, was evaluated by Alamar blue assay. Data are expressed as A.U. of MTT test and of resazurin reduction of each HTMC cultures, and represent the mean ± S.D of 3 separated experiments, in triplicate. *p <0.05 treated vs respective untreated cultures;°p<0.05,°°p<0.01 untreated 48 and 72 hrs vs untreated HTMCs 24 hrs; ^@@^p<0.01 untreated 72 hrs vs untreated HTMCs 48 hrs; +p<0.01 treated 48 and 72 hrs vs treated HTMCs 24 hrs; ^a^p<0.05, ^aa^p<0.01; ^aaa^p<0.001 treated 72 hrs vs treated HTMCs 48 hrs. (Two-way ANOVA followed by Bonferroni posttest).

MTT assay was carried out as a ‘gold standard’ to evaluate cell viability by reference to the mitochondrial compartment functionality during chronic stress exposure (Fig 2 panel A). After 24 hrs of experimental treatment, the viability index of H_2_O_2_-treated HTMC revealed an impairment in both culture models, although it was slightly more marked in 3D- than in 2D-cultures. However, during the following experimental time of exposure to oxidative stress, the mitochondrial functionality resulted restored in both 2D- and 3D-HTMC models, probably indicating an adaptive response to H_2_O_2_.

### HTMC metabolic activity

The effects of chronic 500 μM H_2_O_2_ exposure on 2D-and 3D-HTMC were measured at each check point time up to 72 hrs, by Alamar Blue assay (Fig 2 panel B). 2D HTMC exposed to chronic stress reflected the general trend of untreated cultures, with a decrease of resorufin reduction at the end of the experimental treatment. Conversely, untreated 3D HTMC cultures showed a constant increase of metabolic activities and even during 500μM H_2_O_2_ chronic treatment. Overall, at all time points, treated 3D HTMCs evidenced a significant increase of their metabolic state as opposed to control cultures. This increment decreased slightly after 72 hrs of 500 μM H_2_O_2_ treatment, whilst remaining higher than untreated cells.

### Apoptosis array

**Fig 3:**
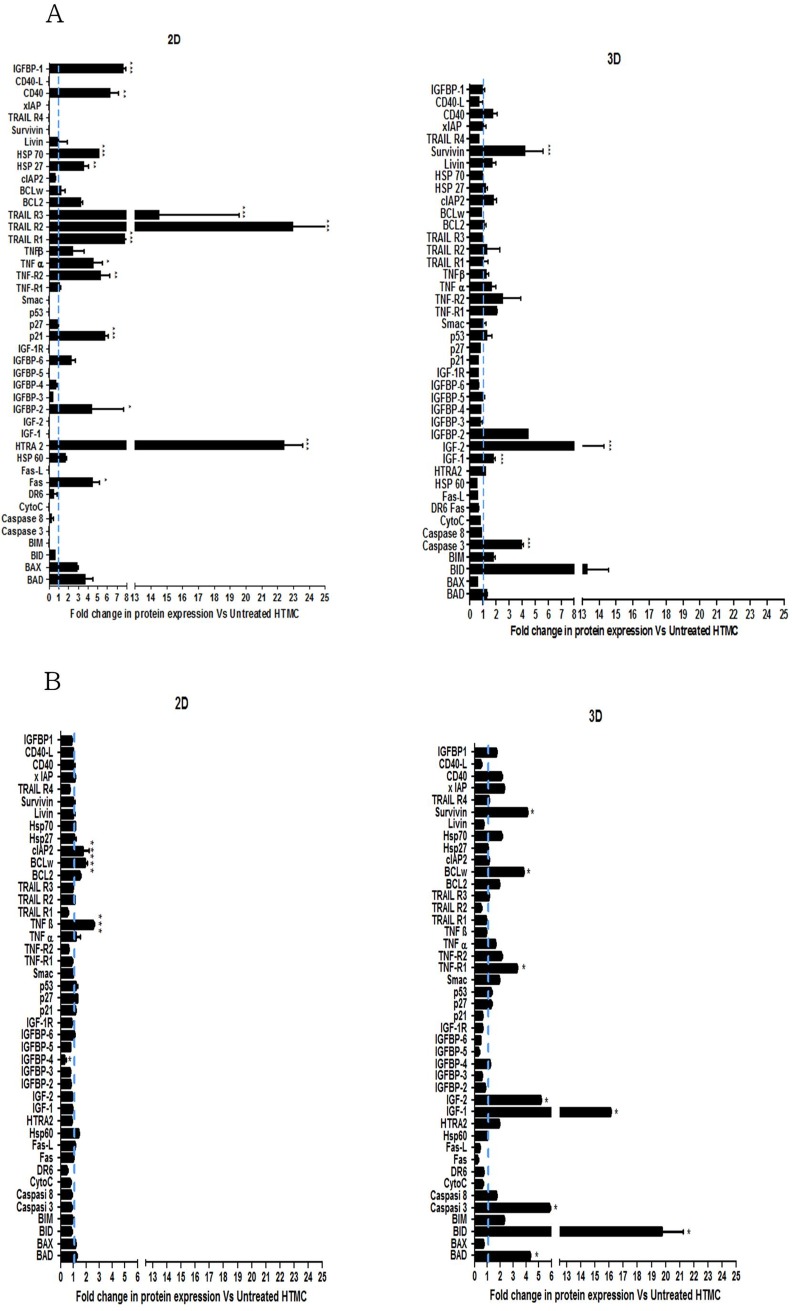
Apoptosis array. Analysis of anti- apoptotic and pro- apoptotic protein levels in 2D- and 3D-HTMC were performed after 48 (panel A) and 72h (panel B) of 500μM H_2_O_2_, by Human Antibody Array C1 (RayBio^®^ C-series). The light blue dotted line represents the protein level of untreated HTMC for each of the 43 proteins examined. For each time point twelve individual models were arrayed (six 2D-HTMC plus six 3D-HTMC) and per experiment the intensity of Positive Control Spot was used to normalize signal responses for comparison of results across multiple arrays. *p<0.05; **p<0.01; ***p<0.001 vs. respective untreated cultures (One-way ANOVA followed by Bonferroni posttest).

Pro- and Anti- apoptotic protein levels were analysed by Human Antibody Array C1 (RayBio^®^ C-Series) using 43 different antibodies (Fig 3). The array patterns highlighted the differences between the 2D- and 3D-HTMC model, in apoptosis ignition. In particular, 3D-HTMCs showed a high throughput profiling in response to the hydrogen peroxide, compared to the 2D model.

### Pro-inflammatory cytokine gene expression analysis

**Fig 4:**
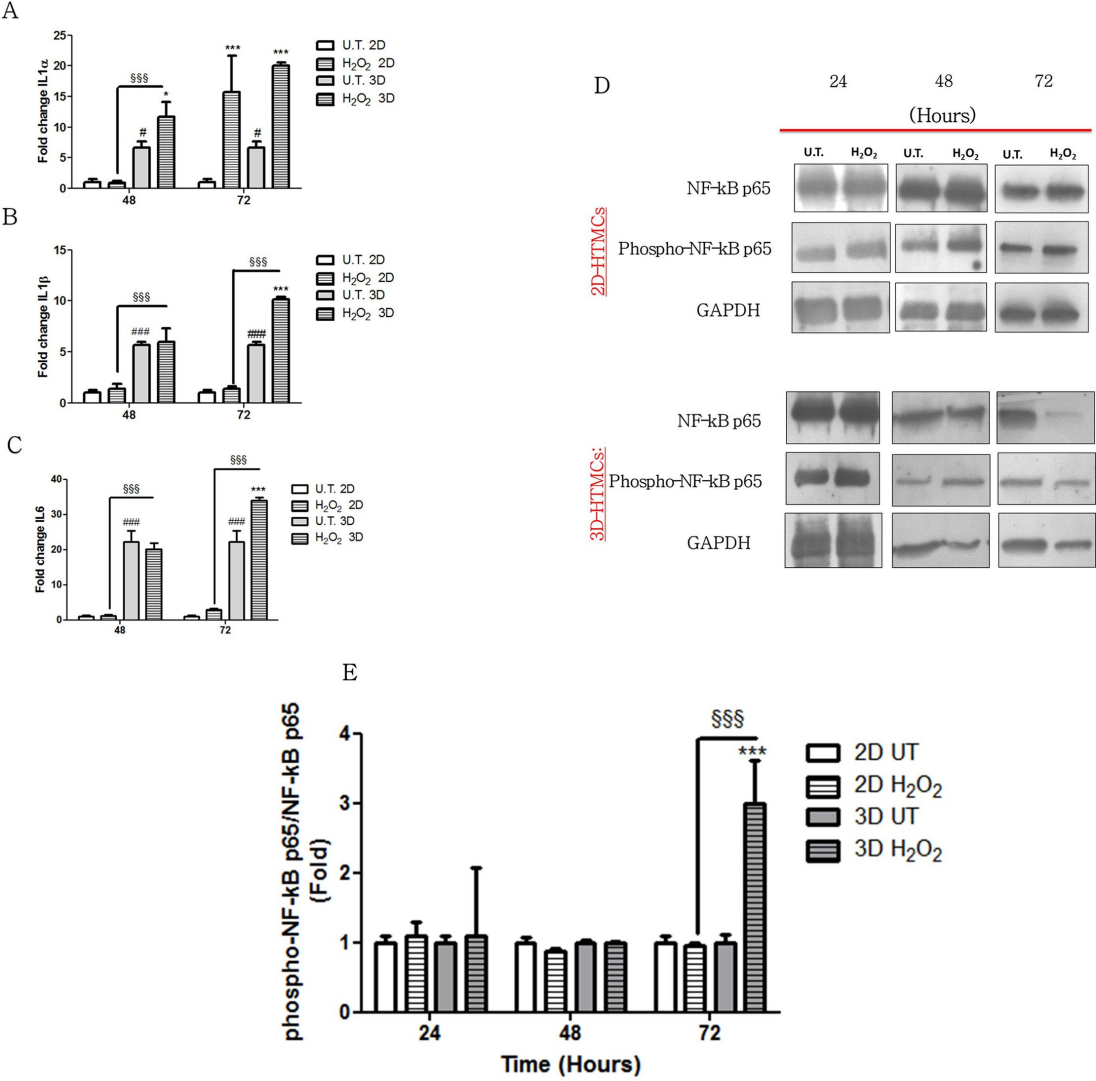
Induction of pro inflammatory factors by chronic H_2_O_2_ treatment. Quantitative PCR gene expression analysis of 2D- and 3D-HTMC subjected 500μM for 48 h and 72 h. IL-1α IL-1β, and IL-6 (Panels A, B and C, respectively). Data are expressed as fold-increase relative to the 2D control at the same end-point and normalized to Ubiquitin housekeeping gene expression. Each bar represents the mean ± S.D. of three independent experiments performed in triplicate. (Panel D) The figures depicted are representative of at least three similar immunoblot analysis of NF-kB (p65), p-NF-kB (p65) protein levels in untreated HTMCs and treated HTMCs (H_2_O_2_) whole protein lysates at indicated time points. GAPDH was used as an internal control for equal protein loading on the gel. (Panel E) NF-kBp65 activation was evaluated in HTMC cells subjected to chronic treatment with H_2_O_2_ for 24, 48 and 72 hrs. The analysis was performed by immunoblotting and the bars represent the ratio of phosfoNF-kBp65/NF-kBp65, and are expressed as fold vs. untreated HTMC cultures. Data represent the mean ± S.D. of 3 independent experiments. ***p <0.001,* p <0.05 treated 3D-HTMCs vs. untreated 3D-HTMCs cells; ### p<0.001, # p <0.05 untreated 3D-HTMC vs. untreated 2D-HTMC cells; §§§p<0.001 treated 3D-HTMC vs. treated 2D-HTMC cells (Two-way ANOVA followed by Bonferroni posttests).

In order to evaluate the pro inflammatory effect of H_2_O_2_ on 2D- and 3D-HTMCs, the cells were treated as mentioned above for 48 and 72 h prior to performing inflammatory gene expression profiling. The gene expression levels of IL-1α, IL-1β and IL-6 were analysed by qPCR (Fig 4 Panels A, B and C, respectively). At 48 hrs, untreated HTMCs cultured in 2D showed a decreased basal gene expression of all three analysed compared to HTMCs cultured in 3D conditions [24,25]. Moreover, at this time point, only treated 3D-HTMCs revealed a slight, but significant increase of IL1α level.

Conversely, at 72 hrs treated 2D-HTMCs showed a marked increase of IL1α, of about 15 fold compared to untreated 2D-cultures, while in 3D-HTMCs an increase of IL1α, IL1β and IL6 of about 3, 2 and 1.5 fold respectively, was observed compared to the untreated 3D-HTMCs.

### NF-kB transactivation after chronic H_2_O_2_ treatment

The level of activated NF-kB was detected by a specific kit containing antibodies against both NF-kB p65 and the phospho-NF-kB p65 subunit to check the inflammatory / antiapoptotic response activation.

2D- and 3D-HTMCs were treated for 24-48-72 hrs as mentioned above and the NF-kB activation was analysed in terms of the ratio between the levels of phospho-NF-kB p65/ NF-kB p65 (Fig 4 panel D). Phospho-NF-kB p65 protein content was up-regulated by H_2_O_2_ treatment (Fig 4 panel E) only in 3D-HTMCs (p<0.001) when compared to both the untreated culture models and the treated 2D-models.

## Discussion

3D cell culture techniques are frequently used to provide a faithful model able to reproduce the microenvironment and cellular responses found in vivo. Here, we analysed the cellular behaviour of commercial human TM from a healthy donor, as provided by the manufacturer (see added information) in order to setup the optimal culture conditions to mimic the in vivo microenvironment during reiterated exposure to oxidative stress since this is considered one of the main risk factors for glaucoma onset. Therefore, the present work aims to compare 2D- and 3D-HTMC models in order to define the best culture conditions for the preliminary performance assessment of an in vitro relevant platform suitable to investigate the early steps of triggering TM damage, an event that is recognized as eventually leading to glaucoma.

In our first approach, confocal microscopy analysis demonstrated that the ECM components contained in Matrigel^®^ allowed the development of the complex architecture of 3D-HTMC in terms of dimension and cell-to-cell contact compared to the 2D-HTMC. Indeed, the latter showed a more confused pattern with less definition of both F-actin and nuclear shape due to the 2D-flattening of the cells typical of a bi-dimensional culture condition (Fig 1 panel A).

As far as the cellular response to oxidative stress (OS) is concerned, we initially investigated the intracellular ROS generation, following H_2_O_2_ exposure, in a time course experiment. The DCF assay confirmed that the impact of OS on the 3D model, which better maintained the spatial architecture of a natural tissue, was averagely greater by about 142% than in the 2D culture, thus indicating that, in a more physiological setup, 3D in vitro cultured HTMC show a higher sensitivity to OS, responding to the extracellular oxidative challenge with a significantly higher intracellular respiratory burst compared to traditional 2D culture conditions (Fig 1 panel B). In our experimental model, both the exposure time of the cells to the oxidant and the shielding effect of Matrigel have been addressed. In particular, it is known that being H_2_O_2_ a small, non-charged molecule, it rapidly spreads in aqueous solutions and easily crosses cell membranes where, in presence of redox-active metal ions, it produces the reactive hydroxyl radical (OH•), which indeed is considered responsible for cytotoxicity [26]. In our experimental setting, a 2 hr exposure time has been chosen because, as it has already been reported [18,19], H_2_O_2_ half-life in presence of cells is quite short (~1 hour), as it is rapidly internalised through the cell membranes and detoxified by intracellular enzymes or converted to the already mentioned reactive hydroxyl radical. Thus, in the 2 hr exposure time the extracellular availability of the oxidant in the cell medium is in any case significantly depleted, as reported by others, and its cytotoxic/pro-inflammatory effect, if any, is likely to have already been exerted on cells. In fact, as already demonstrated by Kaczara et al 2015 [19] there is no difference in terms of cytotoxicity exerted by H_2_O_2_ administered for 2 hrs, changing the cell medium afterwards, or administered without changing the cell medium for 24 hrs. The rate of cell death indeed being unchanged. Thus, supported by the literature, we believe that our experimental setting is the most appropriate to induce a reiterated, sub-toxic effect to HTMC as proposed in our rationale. Indeed, even after H_2_O_2_ removal from the cell medium at 2 hrs, both in the traditional 2D TM setting as well as in the 3D TM model, proposed by us, the intracellular ROS production after 4 hrs is still significantly higher than, and approximately twice as much as, the control, untreated cells (Fig 1 panel B), indicating a long-lasting effect of H_2_O_2_ on the cells, that will likely be reflected on signal transduction. So, we can infer that H_2_O_2_ is able to penetrate and spread in the Matrigel in the same measure as in standard cellular medium and that cells embedded in the Matrigel even become more sensible to H_2_O_2_ challenging, compared to the cells plated in monolayers in traditional cultures. This also explains the significant overall responses in terms of signal transduction, gene expression and cytokine production of HTMC in the 3D setting compared to the 2D.

In this regard, given the evidence that the exogenously-administered H_2_O_2_, depending on its time/concentration, triggers apoptosis in numerous mammalian cells [27], we firstly analysed its effects on the HTMC mitochondrial function and their health state as litmus test of the daily ROS production [28,29]. Therefore, the MTT assay, reflecting succinate dehydrogenase activity, showed a time-dependent reduction of mitochondrial functionality only in 2D-HMTCs while, in 3D-HMTCs, a decrease of such activity was observed only during the first 24 hrs, while in the following period of pulsed stress, an adaptation phenomenon was observed with a homeostasis restoration.

In addition, the cell health state was also analysed by Alamar Blue assay that evidenced a higher metabolic activity in the 3D than in the 2D model, also after prolonged pro-oxidant stimulus. These findings suggest that 3D-HTMCs are able to undergo an adaptation process, probably supported by the ECM protective effect (Fig 2 panels A and B), closer to the in vivo conditions because, given that the chronic effects of glaucoma manifest only over time, it is conceivable that the first signs of oxidative damage to TM are counterbalanced via adaptation pathways.

Thus, the apoptosis array revealed that HTMCs, cultured in 3D culture conditions, were more adept at counteracting the activation of pro-apoptotic proteins in response to daily H_2_O_2_ exposure, than the 2D model (Fig 3 panels A and B). In fact, the significant increase of anti-apoptotic proteins such as Survivin, the pro-survival member of the Bcl-2 family, IGF1 and IGF2, was likely to sustain cell viability through apoptosis inhibition as already reported [30–34], despite the increase of caspase 3, BID and BAD. Moreover, the increase in TNFR1 suggested to us that also the pro-survival NF-kB transcription factor could be involved in this resistance to apoptosis [35].

In the 2D model, the apoptosis pathway was activated after 48 hrs of H_2_O_2_ exposure. Thus, a marked increase of pro-apoptotic proteins such as HTRA2, TRAIL 1–3, FAS was observed, compared to untreated cell cultures, but the anti-apoptotic proteins, able to counteract this cell death activity, did not increase in a significant way [36]. However, some anti-apoptotic protein activation was detectable at 72 hrs, suggesting that, with significant delay respect to the 3D model, the apoptotic pathway finally relented in favour of cell adaptation also in this case.

Further experiments were carried out evaluating both the NF-kB activation over time (Fig 4 panel E), as a crucial trigger of inflammatory pathways, and the gene transcription of pro-inflammatory cytokines including IL-1α, IL-1β and IL6 (Fig 4 panels A, B and C) [37–39].

Thus, since the exogenous OS in our experimental model came in pulses, at 48 hrs of exposure the parallel activation of phospho-NF-kB p65 and the expression of the inflammatory genes it controlled were not yet detectable (Fig 4 panels A,B,C and E). Indeed, a sustained NF-kB activation in the first 48 hrs was not observed in both culture models because during the first two H_2_O_2_ pulses (at time 0 and at 24 hrs) NF-kB activation was perhaps only temporary, with no memory of the previous challenges and no signal accumulation visible by WB [40]. But the situation changed at 72 hrs exposure, and only in the 3D model, where the phospho-NF-kB subunits showed a marked and significant increase (p<0.001) compared to the control untreated cells and also to the 2D setting. These effects suggested that, while in the 2D model the HTMCs did not retain memory of the previous OS pulses even at 72 hrs, in the 3D model, at the same time point, a NF-kB activation memory has been established, likely to be pulling the trigger to the onset of a chronic inflammatory response in the HTM cells like observed in vivo.

Moreover, at 48 hrs, in the 3D model only a slight, but significant, increase of IL1α gene transcription was observed compared to the untreated 3D-HTMCs (p<0.05), corroborating the hypothesis that the cells, during the first two OS pulses, initially responded with a mild and temporary activation of the inflammatory pathway [40]. Conversely, at 72 hrs all three cytokines tested in the 3D model (IL1α, IL1β and IL6) were significantly up-regulated, correlating this result with the corresponding NF-kB sustained activation at this time point, which in turn correlates with the triggering of anti-apoptotic pathways and augmented TM viability in the 3D setting.

In contrast, the HTMCs cultured in 2D did not evidence any sustained activation of such transcriptional factor, not even after 72 hrs of H_2_O_2_ exposure. Nevertheless, the significant increase in IL1α levels, at this time point, leads us to assume that it may depend on the temporary, but not sustained, NF-kB nuclear translocation immediately after the H_2_O_2_ exposure, that is anyway sufficient to trigger some inflammation also the 2D model [41].

Overall, considering the results obtained, we suggest that our 3D-HTMC model may be used as a starting point to study the onset of glaucoma, since it physiologically resembles more in vivo TM features. Furthermore, the HTM-matrix embedded cells seem more sensitive to the OS, engaging what seems to be the onset of a chronic stress state already at 72 hrs of pulsed H_2_O_2_ treatments. Indeed, we demonstrate that, in our 3D model, HTM cells can be studied during exposure to prolonged oxidative stress, which is well known to play a pivotal role in the degeneration of TM, sustaining the neuroinflammatory and neurodegenerative outcomes of glaucoma. Our approach for assessing a human-based in vitro glaucoma model may provide a useful tool also to analyze and check the effectiveness of targeted therapies focused on this complex disease involved in irreversible optical damage.

## Supporting information

S1 File Cell Application Inc. further information. Myocilin expression in HTMC (Cell Application INC.) after Dexamethasone treatment. (PDF)
