# Supplementary material for: 2D- and 3D-cultures of human trabecular meshwork cells: A preliminary assessment of an in vitro model for glaucoma study
Source: PLoS One. 2019 Sep 6;14(9):e0221942. doi: 10.1371/journal.pone.0221942 (PMC6731014; doi:10.1371/journal.pone.0221942)
Supplement: S1 File — (PDF) [file pone.0221942.s001.pdf]

CELL
APPLICATIONS, INC.

Myocilin expression in Trabecular Meshwork Cells was analyzed following dexamathone treatment. After cells reach 70-80% confluency, HTMC cells were treated with 100nM and 500nM of dexamethasone for 6 days in DMEM with 1% FBS. Cell lysates were analyzed for Myocilin and beta-Actin expression by Western Blot.

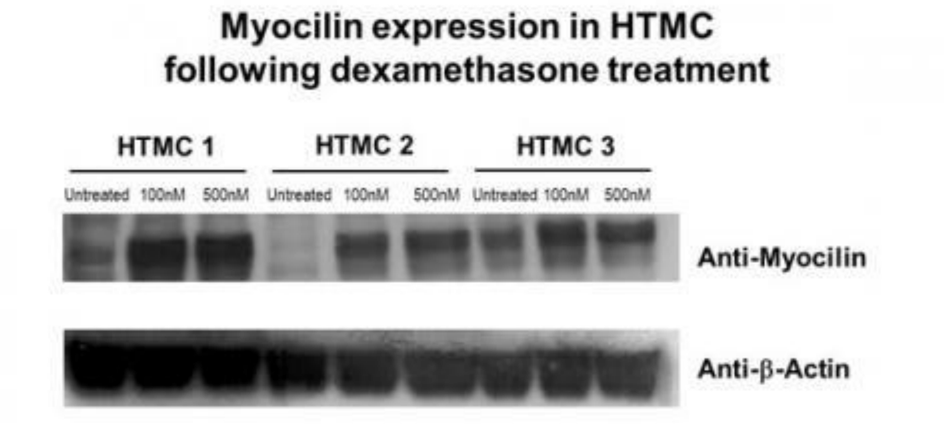

5820 Oberlin Drive #101, San Diego, CA 92121 ♦ (858) 453-0848 ♦ Fax: (858) 453-2862 ♦ www.cellapplications.com
